# One Model is Not Enough: Ensembles for Isolated Sign Language Recognition

**DOI:** 10.3390/s22135043

**Published:** 2022-07-04

**Authors:** Marek Hrúz, Ivan Gruber, Jakub Kanis, Matyáš Boháček, Miroslav Hlaváč, Zdeněk Krňoul

**Affiliations:** 1 Department of Cybernetics and New Technologies for the Information Society, University of West Bohemia, Technická 8, 301 00 Pilsen, Czech Republic; jkanis@ntis.zcu.cz (J.K.); matyas.bohacek@matsworld.io (M.B.); mhlavac@ntis.zcu.cz (M.H.); zdkrnoul@ntis.zcu.cz (Z.K.); 2 Gymnasium of Johannes Kepler, Parléřova 2/118, 169 00 Prague, Czech Republic

**Keywords:** sign language recognition, CNN, Transformer, ensemble

## Abstract

In this paper, we dive into sign language recognition, focusing on the recognition of isolated signs. The task is defined as a classification problem, where a sequence of frames (i.e., images) is recognized as one of the given sign language glosses. We analyze two appearance-based approaches, I3D and TimeSformer, and one pose-based approach, SPOTER. The appearance-based approaches are trained on a few different data modalities, whereas the performance of SPOTER is evaluated on different types of preprocessing. All the methods are tested on two publicly available datasets: AUTSL and WLASL300. We experiment with ensemble techniques to achieve new state-of-the-art results of 73.84% accuracy on the WLASL300 dataset by using the CMA-ES optimization method to find the best ensemble weight parameters. Furthermore, we present an ensembling technique based on the Transformer model, which we call Neural Ensembler.

## 1. Introduction

Sign languages (SLs) are visual languages that convey meaning through the means of body movement and facial expressions. Semantically, they are on par with spoken languages, but they rely on the visual sensory system and not the auditory sensory system. In this paper, we dive into sign language recognition (SLR) with a focus on isolated SLR. The task is defined as a classification problem, where a sequence of frames (i.e., images) is recognized as one of the given SL glosses. The glosses can be roughly expressed as words representing the semantics of the observed sign. The problem of automatic sign language recognition is a relevant topic addressed not only by the scientific community, but also by the general public. Its solution or at least a partial solution may facilitate communication with the authorities or interaction with others in everyday life for people with hearing impairments.

Early systems were based on per-image feature extraction and hidden Markov model-based recognition of the sequences. This pipeline was inspired by the success of automatic speech recognition. The main problem in SLR was the design of the features that need to be extracted. Although linguistically speaking, the important features were already identified (i.e., hand shape, body movements, and facial expression) it was very hard to design a robust algorithm that would be able to extract them.

With the advent of convolutional neural networks (CNN) [1,2,3], the effort of designing hand-crafted features has been shifted toward the preparation of representative data and learning of the features automatically. Soon, methods combining CNN and HMM [4] or CNN and LSTM [5] emerged in the literature. The next breakthrough was achieved with the 3D CNNs [6], modeling the spatio-temporal features. Nowadays, the Transformer model [7], which achieves state-of-the-art results in natural language processing (NLP) tasks, seems to be worth exploring also in the task of SLR. The capabilities of Transformer model in various computer vision tasks have been already demonstrated, i.e., object detection [8], or image classification [9].

In this work, we analyze appearance-based approaches versus pose-based approaches and combine them by an ensembler to obtain the best results. Appearance-based methods are implemented in an end-to-end fashion, where the spatio-temporal features are modeled directly by a neural network, and their classification is performed by the head part of the network. This approach utilizes the raw vision data (for example, RGB images) which comes with some advantages and some disadvantages. The advantage is that the appearance of the hands, face, and body can be observed by the model, which is then able to differentiate between very similar signs. The disadvantage is the relatively larger variability of the data and limited options when it comes to augmentations. The larger variability of appearance implies a need for a larger training set, which is opposed by the limits posed on the augmentation techniques, which are restricted to geometric and brightness transformations.

On the other hand, the pose-based methods are looking only at the per-frame poses of the performers, which automatically suppresses the effect of different environments, clothes, skin tones, and all other appearance-based factors. This means that a lower number of training data might be sufficient for the model to learn from, but some nuances distinguishing between similar signs might be lost. One advantage of the pose-based methods is the possibility to apply more augmentations by changing the relative position of the individual joints to simulate small perturbations in the motion. Additionally, the pose-based methods are less computationally demanding during inference and are thus more suitable, for example, for mobile devices. In general, when a large amount of training data and computational power is available, the appearance-based methods outperform the pose-based methods. If the conditions are not met, one should utilize the pose-based approach, as was shown, for example, in [10].

Our main contributions include a comprehensive study of appearance- and pose-based methods on two well-known datasets, the introduction of a neural ensembler, and achieving new state-of-the-art results on the WLASL300 dataset by using the CMA-ES method, which estimates optimal weighting parameters of an ensemble.

## 2. Related Work

### 2.1. Sign Language Recognition

There are two main streams of approaches in SLR-appearance-based and pose-based. Appearance-based approaches extract unitary representations from the input frames, which can be further used for recognition. The first appearance-based approaches in SLR used shallow statistical modeling, such as hidden Markov models [11,12,13], and handcrafted features [14,15]; however, these approaches performed well only on datasets with a small number of glosses. In the last years, deep learning-based approaches, neural networks especially, have been becoming more and more popular in SLR. Modern appearance-based approaches can be divided into a few categories based on the type of neural network they are utilizing. The first category is based on CNNs [16,17,18,19]. The second category uses recurrent neural networks (RNNs)-namely long short-term memory networks (LSTMs) [20,21]. The third main category is Transformers [18,22]. The last category contains approaches based on 3D CNN originally used for action recognition; however, their use for SLR has also been studied [23,24,25].

With the advances in pose estimation, pose-based approaches have emerged, making use of signer pose representations as the input. Unlike the previous methods, these models do not process raw RGB/RGB-D data, but rather pose representations of the estimated body, hand, and face landmarks. Inspired by Yan et al. [26], who used a spatio-temporal graph convolutional network (GCN) for action recognition, Vázquez-Enríquez et al. [27] used GCNs also for SLR. Last year, Boháček et al. [10] introduced pose-based transformer SPOTER and reached very promising results. In small training data protocol, SPOTER outperforms even the visual-based approaches.

### 2.2. Ensemblement

Ensemble techniques are a widely implemented approach in machine learning [28,29,30]. The diversity within the ensemble is given by data sampling or by modifying the structure of the constituent models. If we assume significant diversity among the individual models, such multiple learning algorithms have a better predictive performance than the single learning algorithms due to minimizing the residuals of the final ensemble. The diversity is explicitly promoted in the error function of individuals. The ensemble model actually minimizes the combination of errors of the individuals by negative correlation learning ensemble [30]. These are mostly known as ensemble models researched on classification tasks. In [31], the effect of ensemble components and ensemble cardinality in a regression task at several levels increases the estimation accuracy of the egocentric hand pose estimation task.

In [32], the effect of both the CNN- and HMM-structure on the hybrid approach is introduced, and a log-linear combination of multiple models further improves performance. The effect of out-of-domain data and ensembles of CNN-HMMs in model combination are investigated to boost performance as a pretrained network prior to finetuning on in-domain data. Model ensembles, combining the raw visual data with the pose estimates, are also used. Jiang et al. [33] combine predictions of pose-based GCN with the results of multiple 3D-CNNs on both RGB data and depth flow.

## 3. Data

In this section, we describe the datasets that we used throughout the experiments and the pipeline that was use to produce the training data for the models.

### 3.1. Datasets

In this work, we conduct experiments on two datasets-AUTSL [34] and WLASL [25]. They represent two types of datasets. AUTSL is focused on more repetitions of a smaller number of signs by a relatively small sample of signers in a “well-behaved” environment, whereas WLASL focuses on the diversity of the signers and the environment. Moreover, WLASL recordings capture expert performers, whereas AUTSL is performed by a mixture of professional and amateur signers. Furthermore, the WLASL dataset is divided into several sub-sets based on the number of glosses to be recognized. In this work, we use the WLASL300 subset. The features of the datasets are summarized in Table 1.

AUTSL captures 43 different signers; 6 of them are Turkish sign language (TSL) instructors, 3 are TSL translators, 1 is deaf, 1 is a child of deaf adults, 25 are TSL course students and 7 are trained signers who learned the signs from the dataset. Ten of these signers are men and thirty-three are women; and also, two of the signers are left-handed. The ages of the signers range from 19 to 50, and the average age of all signers is 31.

The sign instances contained in the WLASL dataset are always performed by native American SL signers or interpreters. The data were collected from multiple public resources intended primarily for the teaching of SL, thus unrestricted varieties of signing styles or dialects, as well as video backgrounds are present.

### 3.2. Data Processing

**Figure 1:**
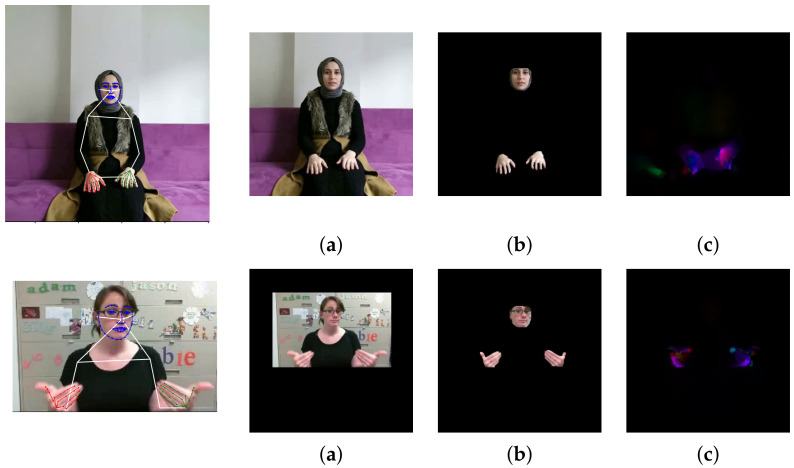
The first line: AUTSL dataset, the second line: WLASL300 dataset. On the left: Positions of joints and salient points on the face in a source RGB video frame of a given size, on the right: Data normalization and preprocessing (**a**) *Crop&Resize* data variant, (**b**) the *Masked* data variant, and (**c**) the *OptFlow* data variant.

Our SLR methods utilize different types of input data (different data modalities) as seen in Figure 1. In this section, we describe all the necessary preprocessing steps to obtain these data modalities from the original RGB images. In the experiments, we consider the following types of data based on the specific preprocessing: *Skeleton* (body pose), *Crop&Resize*, *Masked*, and *OptFlow*.

For creation of the *Skeleton* data variant, we utilize two state-of-the-art frameworks, OpenPose [35] and MMpose [36], to estimate 2D information (x,y) about the positions of joints and salient points (keypoints) on the face in a given RGB image. Both of these pose extractors use deep neural networks that are capable of estimating a complex pose as a skeleton consisting of the positions of the joints of the arms, legs, head, and body, and keypoints on the face; see Figure 1. Both of the extractors also provide confidence for each of the detected points. For OpenPose, we use the $BODY_{25}$ model, which outputs 18 joints for the body, 21 points for each hand, and 69 points on the face. The MMPose model (DARK [37] with HRNet [38] backbone) provides 23 body joints, 21 points for each hand, and 68 face points. In this work, we omit leg joints because they do not carry any discriminative features for our task.

The *Crop&Resize* data variant provides normalization of each frame of the video sequence. Normalization is defined as a transformation that unifies the scale and determines the resulting size of the image data. We also want to respect the signing space, which is a loosely defined space in front of the signer where the signing is happening. This space starts some way above the head, spans the whole upper body, and stops below the waist. It stretches as far as loosely spread arms. For this purpose, we firstly compute skeletal data. The scale of the signer’s body $w_{org}$ is given by the Euclidean distance of the joints of the signer’s left and right shoulder. This distance is more or less constant during the recording of a sign. Next, we define the center (origin) $p_{origin}$ of the signing space. We identify the position of the joint defining the center of the shoulders (OpenPose) or compute this joint as the geometric mean of the shoulder joints (MMpose). This gives us the *X* coordinate of the $p_{origin}$ and we move it lower by $t_{y}\times w_{org}$ pixels. The span of the space is defined as $range_{x}$ and $range_{y}$, which are again derived from $w_{org}$ as its multiplications. These parameters define a 2D rectangle, which is then resized to the desired resolution of $d_{x}$ and $d_{y}$. In this work, we use a square image, which means that $d_{x}=d_{y}=d_{size}$. To keep the original ratio, we also use $range_{x}=range_{y}=range$. The transformation parameters are determined from the first frame of the video sequence where a pose is detected and the calculated transformation is applied to all subsequent frames of the video. Even though this procedure does not take into account moving performers or dynamic changes in the viewpoint, it is sufficient in regard to the datasets that we use.

The *Masked* data variant is based on the *Crop and Resize* variant; however, on top of the previous preprocessing pipeline, we also remove the video background. The binary mask *M* is created by rendering the detected hand skeleton and face region into a black image approximating the areas of the corresponding joints or salient points. These areas are given by the skeleton definition, as seen in Figure 1. The mask *M*, initialized this way, is then post-processed by a 3×3 dilatation repeated *n* times. The mask *M* is created for each frame of the video sequence separately. Multiplication with the original frames produces a frame with the background removed but preserving the most important information for the recognition task. The masking is followed by the *Crop and Resize* normalization in the same way as for the unmasked version of the data.

The last data variant is *OptFlow*. A successful approach to calculating the optical flow between two frames is based on total variation regularization and the robust L1 norm in the data fidelity term [39]. In our experiments, we assume 3× scales to create the pyramid of images with a downsampling factor of two. Next, we set a 3× iterative warping approach to compensate for image nonlinearities. We use 300 iterations per frame in the numerical scheme as a stopping criterion.

## 4. Classification Methods

In this section, we describe the individual classification methods, which were used in this paper. We utilize three types of classification models in total-I3D [6,40], TimesFormer [41], and SPOTER [10]. These specific models were chosen with variability in mind. I3D is representative of CNN-based models, which are considered a gold standard in image classification. TimeSformer represents transformer-based models, which are gaining popularity in the last two years. Lastly, the SPOTER model is another transformer-based model; however, in contrast with the previous two models, which use RGB images as an input, this model uses skeletal data as its input. Hence, I3D and TimeSformer represent the appearance-based methods, while SPOTER represents a pose-based method.

### 4.1. I3D

I3D is a family of convolutional neural network models for video classification. The models are build upon state-of-the-art image classification architectures, but inflates their layers (convolutional, pooling, etc.) from 2D into 3D. This means that all the 2D layers are replaced by their 3D counterparts, while preserving original hyperparameters.

In this work, we use the Resnet50-I3D model, which is based on the very popular architecture Resnet50 [3].

### 4.2. TimeSformer

TimeSformer is a Transformer-based model which utilizes spatiotemporal features from sequences of frames. It takes a sequence of RGB frames as input. The frames are sampled uniformly across the source video. The input is then decomposed into patches the same way as in ViT [9]. The flattened patches are linearly mapped into embedding vectors, which are inputted into the Transformer encoder. TimeSformer consists of *L* encoding blocks with query/key/value vectors computed for each input patch. Self-attention utilizes the softmax activation function. Encoding is obtained as a weighted sum of self-attention coefficients from each head. The vectors are then concatenated and processed by an MLP. The classification consists of MLP with one hidden layer. The authors tested several approaches to space–time attention and claimed that the divided space–time attention provides the best results for Kinetics400 [42] and SSv2 [43] datasets.

### 4.3. SPOTER

SPOTER (sign pose-based Transformer), is based on the Transformer [7] model modified to operate on top of body pose sequences. These are constructed on the frame level by estimating the signer’s pose and applying a linguistics-aware normalization procedure. The flattened pose vectors with added positional encoding flow through the Transformer encoder module. Later, a single classification query enters the Transformer decoder and is decoded into the final class probabilities on top of the decoder module.

We follow the fundamental implementation and training configuration introduced by the authors with two modifications. First, we use either MMPose or OpenPose as the underlying pose estimation library. On top of that, we also include the face meshes (68 or 69 facial landmarks for MMPose and OpenPose, respectively) from these libraries as an additional region in the per-frame skeletal pose representations.

## 5. Ensemble

An ensemble is a machine learning procedure that combines several learning algorithms to obtain better results than the individual procedures are able to achieve. In the context of this paper, we use several types of models (I3D, TimeSformer, and SPOTER) and several data sources (RGB, Masked RGB, Optical Flow, and Skeleton) to train the individual sources for the ensemble. We experiment with two methods, the covariance matrix adaptation evolution strategy, which has shown promising results on the SLR task in the past [44], and we introduce a novel method (a neural ensembler) a Transformer encoder-based model, learning the ensemble from the outputs of the individual models and the ground truth data. A similar approach was adopted in [45] for Bangladeshi sign language while using negative correlation learning ensemble. Lastly, as a baseline, we average the outputs of individual models, i.e., all models are weighted equally.

### 5.1. CMA-ES

In this case, we solve the problem of model weights optimization as a black-box optimization problem. For this purpose, we utilized the CMA-ES (covariance matrix adaptation evolution strategy) [46] optimization algorithm (we choose this method mainly because of its freely available implementation (https://cma-es.github.io/ accessed on 27 June 2022) and solid performance in the range of different optimization problems). It is an evolutionary derivative-free algorithm for difficult non-linear non-convex black-box optimization problems in the continuous domain. We solve for the weights of individual models that are used for computing the weighted average of the models’ decision to produce the final decision.

### 5.2. Neural Ensembler

**Figure 2:**
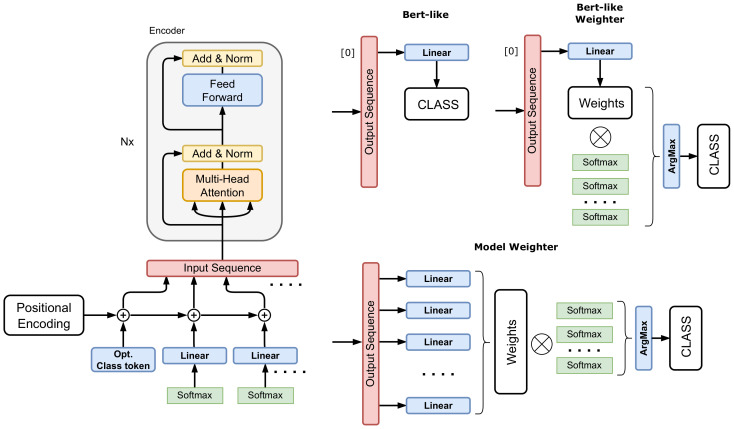
Different types of neural ensemblers. The backbone is a standard Transformer encoder with an optional class token. The rest of the input sequence are the embedded softmax vectors of models to be ensembled for a given video sequence. The output of the encoder has the same length as the input sequence. *Bert-like* decoder uses a class head to predict the final class from the class token. *Bert-like weighter* uses the class token to decode the weights for individual models. *Model weighter*, on the other hand, does not use a class token, but decodes individual sequence elements into a scalar weight that is then used to compute the weighted average.

On top of finding optimal weights for individual models, we experimented with a neural ensembler. It is a trainable ensembler that takes the outputs of individual models and transforms them into a final decision. Thus, the ensemble is not model based but prediction based. This means that the weights for individual models are based on the prediction of one sample rather than the whole set of predictions. In theory, this approach has the disadvantage of potential overfitting, but the advantage is that the ensembler may learn the correlations of the sample predictions of the models. We develop three types of ensemblers based on the Transformer model. The rationale behind using this particular model is the concept of self-attention. In this module, the ensembler can learn the dependencies of the individual model’s predictions. It may even deduce that a prediction with lower confidence may be the right one, based on other models’ predictions. The three proposed architectures are depicted in Figure 2. All the models have the same encoder with the same inputs. The encoder is a standard Transformer encoder with learnable positional encoding. The input sequence consists of the predictions of individual models in the form of the softmax output. These softmax outputs have a dimension equal to the number of classes. They are projected onto the space of the Transformer encoder by a linear layer, and the positional encoding is added to this representation. The sequence flows through several encoder modules and produces a final encoded version of the input sequence. This encoded sequence must be decoded into the final decision. The decoder part is the interesting one that distinguishes the different architectures.

*(1) Bert-like architecture*—a type of decoder that is used in the BERT model [47]. On the input of the encoder, there is a class token representation on the zeroth index. This class token is decoded by using a linear layer with the number of neurons equal to the number of classes. The class with the highest response is considered to be the ensembler decision. This architecture is the one most prone to overfitting since it can produce any result, even such that is not correlated with the original predictions at all. This may occur when all the input models fail to classify a sample correctly, but the ensembler is optimized to produce the ground truth class.

*(2) Bert-like weighter*—instead of producing a decision directly, this model produces a set of weights that are used to weight the inputs to produce the final decision. The dimension of the decoding linear layer is equal to the number of models, and the produced weights are normalized, to sum up to one. The linear layer is connected to the class token of the encoder in a bert-like fashion. Using this setup helps with the overfitting problem. Usually, there is a smaller number of models than classes which means fewer parameters in the decoder and the model only performs re-weighting of the input. Hence, the input has a direct influence on the final decision.

*(3) Model weighter*—is similar to the bert-like weighter, but instead of decoding from the class token, each encoded prediction from each model is decoded by a dedicated linear layer with one output neuron. This neuron predicts the weight for the given model prediction which is then used to re-weight the input. This architecture is a variant of the bert-like weighter, where the class token is omitted and should exhibit similar properties. We wanted to experiment with the added value of the class token.

## 6. Experiments

In this section, we present the experimental setup for each classification model and also for the neural ensembler, the results on the validation and test set, and the discussion.

### 6.1. Data Preprocessing

In this work, we set the parameter of y-shifting the geometric center of shoulders $t_{y}=0.3$, the span of the signing space $range=2\times w_{org}$, and the resolution of the output image $d_{size}=256$. To obtain the masked version of the image, we apply the dilation process two times for the finger joints and the face keypoints, and four times for the wrist joint.

### 6.2. I3D

The implementation of the model is based on [40]. In our final ensembles, we utilized Resnet50-I3D models trained on the three main types of input data: *Crop and Resize*, *Masked*, and *OptFlow*. All the input frames were resized to a size of 256×256 pixels. Sixteen frames from each video were used as an input. The selection of these frames is a pseudo-random choice based on the algorithm from the original implementation. The number of training epochs was set as 80 and 120 on the AUTSL and WLASL300 datasets, respectively. For the parameter update, the SGD optimizer with starting learning rate lr=0.01, cosine learning rate schedule and batch size BS=10 was used. All the I3D models were pretrained on the Kinectics400 dataset.

### 6.3. TimeSformer

We used the official GitHub implementation available from facebookresearch at https://github.com/facebookresearch/TimeSformer, accessed on 27 June 2022. It is implemented using the PyTorch framework [48]. Same as I3D, we trained on three main types of input data: *Crop and Resize*, *Masked*, and *OptFlow*. Since the original paper was trained on the Kinetics dataset and Something-Something-V2 dataset, we had to rewrite the data preparation pipeline and adapt it for AUTSL and WLASL. We increased the number of epochs for training to 30. Other than that, we used the original fine-tuning scenario for divided space–time provided by the authors. The model used for initialization was TimeSformer-K400-8x32-224. The optimizer was SGD with a starting learning rate of 0.005, momentum 0.9, and stepped learning rate decay at epochs (11,21,28) with corresponding steps (0.1,0.01,0.001). The only difference in setup for training between AUTSL and WLASL datasets is the number of classes. The input images were 3-channel RGB. The sampling rate was eight frames per video. We set the batch size to five to better suit the training for our available hardware. For testing, we set the number of ensemble views to one and the number of spatial crops to three.

### 6.4. SPOTER

The SPOTER architecture was implemented using PyTorch. We followed the training setup from the original paper [10]. We hence used the SGD optimizer with a learning rate of 0.001. A scheduler was not employed, and both momentum and weight decay were set to 0. The model’s initial weights were initialized from a uniform distribution within $\left[0,1\right)$.

We also used the same augmentation techniques with a 50% chance of one of them occurring. However, the augmentations’ parameters were found using the Sweep functionality (hyperparameter search) from the Weights and Biases library [49]. We employed its ’Bayesian hyperparameter search’ over 300 runs with smaller training and validation set splits. To find out more about the specific parameters of the augmentation methods affected, we refer the reader to SPOTER’s publication [10].

We trained SPOTER for 100 epochs on WLASL300. On the AUTSL dataset, we trained SPOTER with OpenPose and MMPose for 12 and 35 epochs, respectively, as each model variant was trained until it converged on the training split.

### 6.5. CMA-ES

We computed the weights of the ensemble. The inputs into the algorithm are the predicted softmax or logits output values of particular models from the validation set, whereas the algorithm should maximize the validation set recognition rate. Given the stochastic nature of the method, we ran the optimization process multiple times (6 independent runs) with a randomly weighted ensemble as a starting point and chose the best-performing weights. Generally, as CMA-ES does not require a tedious parameter tuning for its application thus, we set the parameters based on heuristics: sigma0 to 0.25, number of restarts to 3 (restarts), restart from the best to false (restart_from_best) and the parameter bipop to true. All other parameters were in default.

### 6.6. Neural Ensembler

The neural ensembler was implemented in PyTorch. It has a sequence of softmax vectors on the input. Each vector represent one output of one model. The length of the sequence is equal to the number of models that are being ensembled. All models use learnable positional embedding that is added to the embedded softmax vectors. The position represents the identity of the model that produced the softmax. To find the optimal architecture of the neural ensembler and optimal hyper-parameters, we used a Bayesian hyperparameter search as implemented in the Weights and Biases framework. For each dataset, we searched for the following architectural parameters: *dim_feedforward*—the dimension of the hidden layer in the feed forward module in the encoder, *num_layers*—the number of encoder layers (Nx), *num_heads*—number of heads in the self-attention module, and *num_per_head*—dimensionality of each head (num_heads×num_per_head = the model dimension). The hyper-parameter search considers *learning_rate*, *optimizer* (either SGD with momentum 0.9 or Adam), and augmentation values. The augmentation of the input data is two-fold. (1) We add a Gaussian noise with zero mean and we search for an optimal value of the variance. Afterward, the augmented softmax vector is normalized to unit magnitude. This simulates small perturbations in the output of the individual models and mainly should help to overcome the overfitting of the ensembler. (2) We simulate a total uncertainty of a model by setting all the values of the softmax vector to 1/N, where *N* is the number of predicted classes. This way, we want to force the ensembler to not copy the decisions of only a few models, but focus on the whole ensemble. We search for an optimal value of the probability of application of this procedure to individual softmax values. The results can be seen in Table 2.

For the AUTSL dataset, we have 14 trained models (see Table 3) available, whereas for WLASL there are 13 trained models available. The softmax outputs of these models form the input sequences for the the neural ensembler. To train the ensembler, we use the validation data of the datasets and test them on the test data. Thus, for AUSL, there are 4418 training samples and 3742 testing samples. For WLASL, there are 900 training samples and 666 testing samples. The batch size was set to 32. By observing a few runs of the training procedure, we opted for setting a stopping condition of the training to the maximal number of epochs, where the training loss stabilizes for several epochs. This is also motivated by the fact that we do not have any validation data. For AUTSL, where a lot more training data are available, the maximum number of epochs was set to 40, and for WLASL it was set to 600. In different tables, we report the results from the last epoch, even if it is not the best test result.

### 6.7. AUTSL

We trained 14 models on the AUTSL dataset in total-5 I3D models, 3 TimeSformer models, and 6 SPOTER models, and their results can be found in Table 3. The AUTSL dataset was preprocessed by both OpenPose and MMPose. I3D reached comparable results independently on the preprocessing type; however, SPOTER performed significantly better while using MMPose skeletonization. For more details, see Section 7.3. Among single models, I3D trained on *Crop and Resize*-MMPose data reached the best results of 92.86%. While using the classic ensemble method (Ens. OPT), we improved the results by an additional 3.18–96.04%. Neural Ens. (Bert) reached our best result 96.37%, which is slightly worse than the current state-of-the-art method [33]. We can see that I3D models outperformed the Transformer-based models by a large margin. This hints at the observation in other works, that Transformers generally need much more data to be as successful or better than CNNs. This has been experimentally shown, for example, in the ViT paper [9]. Another interesting observation is that I3D performed the best on data that are not masked and contain clutter, whereas the TimeSformer model performed better on masked data. Furthermore, even the optical flow data seems to yield better results. This again might be given by the need of the Transformer for more data. When presented with data without the clutter the relatively low amount of them describes the distribution more densely.

When looking at the ensemble results it is worth mentioning, that even a simple average of the softmax or logits produced by the models leads to a better result than the best individual result (95.80% vs. 92.86%). The absolute increase in accuracy of 2.94% is not negligible and it shows that the models can help each other in the decision making. When the weights are further optimized by the CMA-ES algorithm, the accuracy is increased even more by 0.24%. It is interesting that some weights were found as negative. We did not restrict the search to the positive sub-space and the optimal value was found indeed in the negative part of the weight space (but all weights still have to sum to one). Mathematically speaking, the negative weight means that the decision of the model should be looked upon inversely. The high response for a given class is subtracted from the ensemble decision. We observe this behavior both in the experiment with softmax and also with logits. For us, it is an unexpected result, and it might be interesting to look into in more detail in the future. When we compare the results with softmax versus the results with logits, they are very close together. Interestingly, the equal weights did not result in the same accuracy, meaning that there is more information encoded in the logits than in the normalized softmax values. This is possible due to the models producing logits of an unconstrained magnitude independently of each other. In the weighted averaging scenario, these magnitudes play a role and may result in a discrepancy in the accuracy.

The values of the found weights of individual models show that the distribution of I3D weights is more or less uniform. In the case of TimeSformer, the *Crop and Resize* model seems to be ignored in favor of the masked and optical flow options—the options with less data bandwidth. Most dramatic changes from softmax to logits are observed in the case of the SPOTER model. While in the case of softmax, the MMPose model is weighted very heavily (has the highest weight value in the whole ensemble), in the case of logits, the weight drops rapidly and is distributed more uniformly.

The neural ensembler achieved the best result and is very consistent. We attribute this to the properties of the AUTSL dataset. It seems the neural ensembler performs very well when the individual models are trained highly above average. In this case, the Bert-like model performs the best, since it has the most freedom when making a decision and does not suffer from overfitting since the individual models are very consistent.

Lastly, we tested the hypothetical maximal accuracy of the ensembled models on the validation data. This is denoted as model picker. For each sample, we look at whether at least one model predicted the class correctly. We can see that there are 98.96% of such samples, which is much higher than the best validation score of the CMA-ES ensembler (96.06%). The neural ensembler comes much closer (97.56%) since it is able to make decisions on individual samples. Still, there is some theoretical room for improvement.

### 6.8. WLASL300

On the WLASL300 dataset, we trained 13 models in total—6 I3D models, 6 TimeSformer models, and 1 SPOTER model. For the results, see Table 4. Only MMPose was used for dataset preprocessing since it performed better in the AUTSL experiment. From single models, I3D trained on *Crop and Resize* data using the AUTSL pretraining (see Section 7.1) reached the best results—60.66% once again. From the ensemble methods, the CMA-ES ensemble utilizing logits (Ens. Logits OPT) reached the best results—**73.82**%, which is, to our best knowledge, the new state of the art, which surpasses the previous state of the art of 73.43% [50]. However, it should be noted that the difference is quite small, and further statistical analysis of both approaches could reveal more details.

The results for I3D are in accordance with the observations on the AUTSL dataset, and from this point of view, the I3D model is the most consistent one. It also achieves the best accuracy as the individual model. The TimeSformer model changes its behavior when we consider the large drop in performance on the optical flow data. This may be due to the larger variability of viewpoints when compared to AUTSL. A similar drop is observed with the I3D model, but this drop was expected, given the AUTSL results.

The CMA-ES ensemble works very well on the validation data. The best result of 83.22% is a huge improvement when compared to the best individual effort of 66.78%. On the other hand, the drop in test data is much more dramatic. The same goes for the neural ensembler. The most affected one is the bert-like ensembler, which overfitted on the validation data to an exceptional 99.67% and dropped to the worst result among the neural ensemblers. In the case of less efficient ensembled models, the neural ensemblers with a re-weighting strategy are more potent. Still, they lack the accuracy of the CMA-ES ensemble.

## 7. Ablation Study

### 7.1. Transfer Learning

Usage of pretrained (foundation) models [51] is a common practice in a variety of tasks. In this paper, we investigate the performance of models pretrained on the AUTSL dataset and finetuned on the WLASL300 dataset, and corresponding models trained the other way around. In Table 5, we can see the difference between the original models which were directly trained on the WLASL300 dataset starting from Kinetics400 pretrained weights, and the transferred models which were first fully trained on the AUTSL dataset and then fine-tuned on the WLASL300 dataset. It can be seen that the transferred models reached better results in all cases.

We believe the reason for the performance increase is two-fold. First, it is a well-known fact that neural networks, and Transformers especially, are data hungry; however, the WLASL300 dataset belongs to the category of the small datasets. Therefore, additional training data from the same domain can be beneficial for the final performance. Second, the AUTSL dataset contains a large variety of signs, signers, and also backgrounds. The pretraining on such dataset results in a model with a great ability to generalize containing informative and distinctive features. Models with these proprieties are suitable for transfer learning. To confirm this hypothesis, we also performed the opposite experiment—models fully trained on the WLASL300 dataset were fine-tuned on the AUTSL dataset. Nevertheless, these models reached only comparable results with those directly trained on the AUTSL dataset. We argue that due to the small size of WLASL300, this dataset is not suitable for the model pretraining. This is an important finding since it shows that more repetitions of the same sign by a smaller number of performers are better suited for transfer learning than a dataset with larger variability of performers. Additionally, WLASL300 has more glosses than AUTSL (although comparable) which does not help in transfer learning. This might be useful in the domain of few-shot learning when selecting a suitable pretraining dataset.

### 7.2. Ensemble Transfer

In this experiment, we tried the transferability of optimal ensemble weights found for the given set of models trained on one dataset to other ones trained on the other dataset. Particularly, we used optimal ensemble weights of seven models (I3D *Crop&Resize*-MMPose, I3D *Masked*-MMpose, I3D *OptFlow*, Timesformer *Crop&Resize*-MMPose, Timesformer *Masked*-MMpose, Timesformer *OptFlow*, SPOTER MMPose) trained on the AUTSL dataset in the ensemble of the same type models, but trained on the WLASL300 dataset. The results are 95.54% accuracy on the AUTSL validation data after ensemble weights optimization, which translates to 95.48% accuracy on the AUTSL test data, and after using these weights for the WLASL300 test data, we obtain only 63.51% accuracy, which is worse than 65.02% accuracy for the equally weighted ensemble. Thus, it seems that in the case of different datasets, using an equally weighted ensemble is a safer option than using the weights optimized only on one dataset.

### 7.3. Openpose vs. Mmpose

From a practical point of view, it is interesting to observe the impact of the pose estimation algorithm on the accuracy of the recognizer. In this work, we experimented with two mainstream frameworks for pose estimation—OpenPose [35] and MMPose [36]. OpenPose implements a CNN to predict the probable locations of joints and matches them into the skeletal representation. MMPose implements many algorithms of pose detection, and in this work we use the technique, DARK, introduced in [37] with the HRNet backbone. We performed the experiments on the AUTSL dataset. For the appearance-based methods, we used I3D to test the impact of the pose estimation in the data preprocessing step. The differences are very small, since the pose estimation is used only to determine the crop of the input image, nevertheless MMPose slightly outperforms OpenPose. A more direct impact on the results is expected (and observed) with the pose-based method of SPOTER. It can be seen from Table 3 and Table 6 that MMPose outperforms OpenPose consistently. The margin is substantial, ranging from less than 1% to more than 6%. The only exception is the validation data result for input data without the facial keypoints. In the test phase, MMPose again performs better. Generally, it seems that the quality of detection of the facial keypoints is the most impactful part of the pose detection system. This allows us to conclude that the methods of recognition are generally dependent on the quality of the pose estimation technique and with more robust systems, the recognition can be even better, especially in the case of pose-based recognition.

### 7.4. Facial Keypoints Impact on SPOTER Performance

In Table 6, we summarize the results of SPOTER for different representations of the pose on the AUTSL dataset. The best results are achieved with the full pose, including the facial keypoints. To reduce the dimensionality of the input data, we omitted the metacarpal joints of the hands. These joints should not have a major impact on the recognition. From the results, we see that even though the impact is indeed small, it is not negligible. For MMPose, it is a relative drop of 1.6%, while for the OpenPose, it is 2.4%. In SL, the face encodes part of the non-manual component. Depending on the linguistical richness of the dataset, the impact may vary. In our case, we observe a relative drop in accuracy of 9.1% for MMPose estimations and 2.8% for OpenPose estimations. These results imply that MMPose detects the face more precisely, and the keypoints should be used for recognition.

**Table 6:** Ablation of SPOTER performance on AUTSL depending on included joints. The experiments were performed with two pose estimation libraries (MMPose and OpenPose).

Joint Configuration	Framework	VAL	TEST
All joints and keypoints	MMPose	85.31	84.90
	OpenPose	80.04	78.89
No metacarpal joints	MMPose	83.77	83.51
	OpenPose	78.59	76.96
No face keypoints	MMPose	76.73	77.15
	OpenPose	79.49	76.62

## 8. Conclusions

Isolated sign recognition can still be a very hard problem in an uncontrolled environment, even for modern approaches. In this paper, we analyze the performance of three different methods (I3D, TimeSformer and SPOTER) on two different benchmark datasets (AUTSL and WLASL300). We also analyze the performance of these methods in the context of different input data modalities. Despite the fact that individual models performed sub-optimally, by using the weighted ensemble, we reached very competitive results on the AUTSL dataset (96.37%) and achieved new state-of-the-art results on the WLASL300 dataset (73.87%). In this paper, we also propose a novel neural ensemble method, which we believe has high potential for future research. Moreover, we add an extensive ablation study of the behavior of the individual models. We can conclude that the MMPose framework with DARK and HRNet backbone outperforms the OpenPose algorithm, mainly in the pose-based SLR. Furthermore, the facial landmarks are important to distinguish between signs, and using all hand joints results in the highest accuracy. Any form of ensemble that improves the recognition capabilities of individual models in the task of isolated SLR ensemble is what we need.

## Figures and Tables

**Table 1:** Overview of the datasets. Column “Mean” refers to the average number of video instances per gloss (class).

Dataset	Language	Sensor	Gloss	Videos	Mean	Signers
WLASL300	ASL	RGB	300	5117	17.1	109
AUTSL	TSL	RGB+D	226	38,336	169.6	43

**Table 2:** Optimal settings for neural ensembler.

Model	Data	Dim_ff	Layers	Heads	Per_head	Lr	Optim.	Aug.
BERT-like	AUTSL	504	4	8	18	$4.9\times 10^{-5}$	SGD	
BERT-like		648	5	9	52	$3.5\times 10^{-5}$		✓
W-BERT		472	5	5	38	$7.6\times 10^{-3}$		
W-BERT		572	5	7	20	$5.4\times 10^{-3}$		✓
W-Model		886	4	6	45	$8.6\times 10^{-3}$		
W-Model		973	6	7	37	$9.2\times 10^{-3}$		✓
BERT-like	WLASL	248	5	7	64	$3.9\times 10^{-4}$	SGD	
BERT-like		504	4	8	18	$4.9\times 10^{-5}$		✓
W-BERT		937	4	8	29	$1.0\times 10^{-4}$		✓
W-Model		596	6	8	33	$1.3\times 10^{-3}$		✓

**Table 3:** AUTSL results. Model Picker = percentage of samples that were recognized correctly by at least one model. Ens. EQ = equally weighted ensemble, Ens. OPT = ensemble with optimized weights, Logits = using logits outputs instead of softmax, Neural Ens. = neural ensembler, Bert = bert-like transformer, W-Bert = bert-like weighter, W-Model = model weighter. $W_{Ens.}$ and $W_{Ens.Logits}$ = weights of individual models in the CMA-ES ensemble.

Method	Data	VAL [%]	TEST [%]	$W_{\mathrm{Ens}.}$	$W_{\mathrm{Ens}.\mathrm{Logits}}$
I3D	*Crop&Resize*-OpenPose	**92.87**	92.84	0.10	0.05
	*Masked*-OpenPose	91.90	91.07	0.08	0.08
	*Crop&Resize*-MMPose	92.51	$\underline{92.86}$	0.08	0.15
	*Masked*-MMPose	91.51	91.45	0.13	0.20
	*OptFlow*	88.68	88.00	0.12	0.15
TimeSformer	*Crop&Resize*-MMPose	83.39	81.08	0.03	−0.01
	*Masked*-MMPose	87.55	85.89	0.12	0.09
	*OptFlow*	85.60	82.82	0.10	0.05
SPOTER	MMPose	85.31	84.90	0.23	0.10
	MMPose no Face	76.73	77.15	−0.14	−0.07
	MMPose no iHands	83.77	83.51	−0.0004	0.11
	OpenPose	80.04	78.89	0.01	0.14
	OpenPose no Face	79.49	76.62	0.12	−0.03
	OpenPose no iHands	78.59	76.96	0.02	−0.01
Ens. EQ		95.07	95.80		
Ens. OPT		95.84	$\underline{96.04}$		
Ens. Logits EQ		95.04	95.99		
Ens. Logits OPT		$\underline{96.06}$	95.83		
Neural Ens. (Bert)		97.22	**96.37**		
Neural Ens. (W-Bert)		95.22	95.41		
Neural Ens. (W-Model)		95.31	96.29		
Neural Ens. (Bert) + Aug		$\underline{97.56}$	96.29		
Neural Ens. (W-Bert) + Aug		95.13	96.21		
Neural Ens. (W-Model) + Aug		95.38	96.21		
Model Picker		98.96	—		

**Table 4:** WLASL300 results. Only MMPose is used during the preprocessing step. When we used the fine-tuned models, the results are reported as (WLASL only/AUTSL pretrain). $W_{Ens.}$ and $W_{Ens.Logits}$ = weights of individual models in the CMA-ES ensemble.

Method	Data	VAL [%]	TEST [%]	$W_{\mathrm{Ens}.}$	$W_{\mathrm{Ens}.\mathrm{Logits}}$
I3D	*Crop&Resize*	63.44/**66.78**	55.26/**60.66**	0.10/0.12	0.07/0.12
	*Masked*	61.67/63.00	55.11/57.06	0.06/0.08	0.003/0.12
	*OptFlow*	45.56/50.56	36.19/39.49	0.05/0.04	0.03/0.11
TimeSformer	*Crop&Resize*	43.67/59.00	36.79/52.55	−0.10/0.14	−0.08/0.28
	*Masked*	46.44/58.33	40.84/50.75	0.07/0.10	−0.03/0.08
	*OptFlow*	26.78/48.67	22.37/37.99	−0.16/0.32	−0.26/0.23
SPOTER	MMPose	57.30	53.75	0.19	0.33
Ens. EQ		75.78	66.52		
Ens. OPT		80.33	70.72		
Ens. Logits EQ		77.11	69.52		
Ens. Logits OPT		**83.22**	**73.87**		
Neural Ens. (Bert)		**99.67**	68.92		
NE (W-Bert) + Aug		75.56	69.22		
NE (W-Model) + Aug		75.56	**70.12**		

**Table 5:** Difference in accuracy between the original models and the transferred models (models with the AUTSL pretraining) on WLASL300.

Method	Data	VAL	TEST
I3D	*Crop&Resize*	+3.34	+5.40
	*Masked*	+1.33	+1.95
	*OptFlow*	+5.00	+3.30
TimeSformer	*Crop&Resize*	+15.33	+15.76
	*Masked*	+11.89	+9.91
	*OptFlow*	+21.89	+15.62

## Data Availability

In this work were used two following publicly available datasets: AUTSL—http://cvml.ankara.edu.tr/datasets, accessed on 27 June 2022, and WLASL300–https://dxli94.github.io/WLASL, accessed on 27 June 2022.
